# Spinal Anæsthesia

**Published:** 1901-02

**Authors:** 


### SPINAL ANÆSTHESIA.

THE method of producing anæsthesia by subarachnoid injections of cocaine solution into the spinal canal, suggested by Bier and exploited by Tuffier, has now been employed by so many different observers, who have reported their results, that a fairly reliable conception as to the efficacy of this procedure and as to its dangers may be obtained.

It seems to be well established that an injection of twenty minims of a one per cent. solution of cocaine into the subdural space by means of a needle driven in between the third and fourth lumbar vertebræ will occasion total anæsthesia of the lower extremities, the pelvis, buttocks, and inguinal region for a period varying from thirty to sixty minutes, without interfering with the consciousness of the patient and without producing signs of cocaine poisoning. During this period major operations, such as resection of the knee-joint, amputation, and the radical cure of hernia, may be undertaken and carried out without the patient's experiencing the slightest sensation.

Severneau (*Revue de Chirurgie,* No. 9, 1900) has employed this method of producing anæsthesia in seventy cases, the dose of cocaine varying from one-sixth to one-third of a grain. He notes that although anæsthesia was usually produced, the after-effects were sometimes extremely alarming; profound weakness, pallor, irregularity of heart action, stiffness of the neck, headache, and vomiting were sometimes persistent for two or three days and were obstinate to treatment, though caffeine and an injection of artificial serum seemed helpful. As a rule, these symptoms were but slightly marked and did not last longer than twenty-four hours.

Tuffier notes that, following the injection, anæsthesia begins by a sense of pricking, crawling, and weight in the feet and legs, weight in the epigastrium, some nausea, and, exceptionally, vomiting. In addition, the patients become pale, have flashes of heat, sweat profusely, and the pulse becomes rapid. Headache is nearly always pronounced, but, as a rule, disappears in the night following the operation; sometimes it is agonizing and lasts for days. Tuffier has employed the method in one hundred and twenty-six cases without fatality in so far as the injection of cocaine was concerned.

Racoviceavnu has practised these injections in one hundred and twenty-five cases, failing entirely to produce anæsthesia in four, while in two others the effects were so transitory as to be of no service. In eighty-three cases the after-effects were extremely well marked, though not, as a rule, threatening.

Marx (*Medical News,* August 25, 1900) has employed this method of anæsthesia during labor, and states that it checks almost entirely the pains without the least danger to mother or child. Indeed, the mother does not know the child is delivered till assured of the fact by its cries. Complications of a severe grade were not noted.

Nicoletti, on the basis of an experimental research, maintains that these subarachnoid injections cause no anatomical alterations of the nervous elements.

In making these injections, the skin is thoroughly disinfected as for a surgical operation, the body of the patient is flexed, the spinous process of the fourth lumbar vertebra is outlined (and this is rendered easy by remembering that between the spinous process of the fifth lumbar vertebra and the first sacral vertebra is a pronounced depression), the hypodermic needle, between three and four inches in length, is driven in the middle line—preferably half an inch to one side—upward, forward, and when the lateral puncture is made a little inward, until the escape of some drops of cerebrospinal fluid indicates it has reached the subarachnoid space. Should the point of the needle encounter the bone, it is shifted until the intervertebral space is found. The cocaine solution is then injected and the needle is withdrawn. The procedure is an extremely easy one.

Except for the disagreeable and often threatening after-effects, this method of anæsthesia would in its workings be ideal. These after-effects can scarcely be attributed to the toxic effect of cocaine, and are probably due to a secondary congestion. It would seem wise to substitute for cocaine either eucaine, which may be thoroughly sterilized by boiling and is but one-fifth as toxic, or nirvanin, which is in itself antiseptic and is but one-tenth as toxic as cocaine. It is improbable, however, that this substitution will be followed by sequelæ less marked than those which develop when cocaine is employed in moderate doses, hence it would seem that subarachnoid injections are not likely to replace the already well established and comparatively safe methods now in vogue, save in exceptional circumstances. It is certainly true that until the method has received a more extended trial, and its immediate and remote dangers are better understood, it is not one which should be generally adopted.—*The Therapeutic Gazette.*

